# Dimensional structure of DSM-5 posttraumatic stress symptoms in Spanish trauma victims

**DOI:** 10.3402/ejpt.v7.32078

**Published:** 2016-12-13

**Authors:** Carmen Soberón, María Crespo, María del Mar Gómez-Gutiérrez, Violeta Fernández-Lansac, Cherie Armour

**Affiliations:** 1 Department of Clinical Psychology, Complutense University, Madrid, Spain; 2 Psychology Research Institute, University of Ulster, Coleraine, Northern Ireland

**Keywords:** Posttraumatic stress disorder, DSM-5, trauma, latent structure, confirmatory factor analysis

## Abstract

**Background:**

Confirmatory factor analytic studies have shown that posttraumatic stress disorder (PTSD) symptoms included in the fifth edition of the Diagnostic and Statistical Manual Disorders (DSM-5) may be better explained by two 6-factor models (the Externalizing Behaviours model and the Anhedonia model) and a 7-factor Hybrid model. The latter model comprises the symptom clusters of intrusion, avoidance, negative affect, anhedonia, externalizing behaviours, and anxious and dysphoric arousal. This model has received empirical support mainly in American samples. Of note, there have been a limited number of studies conducted on samples from other countries.

**Objective:**

This study aimed to examine the underlying dimensionality of DSM-5 PTSD symptoms in a Spanish clinical sample exposed to a range of traumatic events.

**Method:**

Participants included 165 adults (78.8% females) seeking treatment in trauma services in the Madrid area (Spain). PTSD was assessed using the Global Assessment of Posttraumatic Stress Scale 5, a Spanish self-report instrument assessing posttraumatic symptoms according to the DSM-5 criteria. Confirmatory factor analyses were conducted in Mplus.

**Results:**

Both the 7-factor Hybrid model and the 6-factor Anhedonia model demonstrated good and equivalent fit to the data.

**Conclusions:**

The findings of this study replicate and extend previous research by providing support for both the 7-factor Hybrid model and the 6-factor Anhedonia model in a clinical sample of Spanish trauma survivors. Given equivalent fit for these two models and the fewer number of latent factors in the Anhedonia model, it was selected as optimal in a traumatized Spanish sample. Implications and future research directions are discussed.

**Highlights of the article:**

- The 7-factor Hybrid model (which comprises the intrusion, avoidance, negative affect, anhedonia, externalizing behaviours, and anxious and dysphoric arousal symptoms clusters) and the 6-factor Anhedonia model (in which the externalizing behaviour symptoms are part of the dysphoric arousal symptom cluster) provided equivalent fit to the data.
- The Anhedonia model is the most parsimonious and thus the optimal-fitting model in the current sample.
- The findings support the distinctiveness between dysphoric arousal, anxious arousal, negative affect, and anhedonia factors.
- The separation of the externalizing behaviour symptoms from the dysphoric arousal symptoms does not improve the model fit in the current sample.

Posttraumatic stress disorder (PTSD) was first introduced into the DSM-III (American Psychiatric Association [APA], 1980) as an official diagnostic category in 1980; since then, there have been many subsequent revisions. The most notable revisions are concerned with (1) the overall number of symptoms comprising the disorder and (2) the number of symptom clusters. The latter has been widely debated, particularly as it pertains to the categorization of the DSM-IV symptomatology for PTSD. The DSM-IV model of PTSD (and the DSM-IV-TR, given no changes to PTSD criteria across these DSM editions) specified three latent factors of re-experiencing, numbing, and hyperarousal. However, a number of alternative models gained a wealth of empirical support across a variety of trauma-exposed populations; these include the 4-factor Emotional Numbing model (King, Leskin, King, & Weathers, 1998; intrusion, avoidance, emotional numbing, hyperarousal), the 4-factor Dysphoria model (Simms, Watson, & Doebbeling, 2002; intrusion, avoidance, dysphoria and alterations in arousal, and reactivity or hyperarousal), and the 5-factor Dysphoric Arousal model (Elhai et al., 2011; intrusion, avoidance, numbing, dysphoric arousal, and anxious arousal). The latter model split the hyperarousal symptoms into two separate factors: dysphoric arousal (sleep difficulties, anger and irritability, and concentration difficulties) and anxious arousal (hypervigilance and exaggerated startle response) factors (Watson, 2005). This body of literature can be perused in a recent comprehensive systematic review on the topic (cf. Armour, Műllerová, & Elhai, 2016).

The most recent edition of the DSM, the DSM-5 (APA, 2013), characterizes PTSD as being consisted of 20 symptoms, each belonging to one of four symptom clusters: intrusion (I), avoidance (A), negative alterations in cognitions and mood (NACM), and alterations in arousal and reactivity (AAR). Shortly after the release of the DSM-5, two research teams presented alternative models comprising six symptom clusters. Both models are an extension of the 5-factor Dysphoric Arousal model. The 6-factor Anhedonia model (Liu et al., 2014) splits the NACM factor into negative affect and anhedonia (i.e., reduced positive affect), thus taking into account the theoretical and empirical evidence suggesting that negative and positive affects are distinct constructs (Watson, 2005, 2009; Watson, Clark, & Stasik, 2011). The 6-factor Externalizing Behaviours model (Tsai et al., 2014) included the externalizing behaviours factor that comprises the E1 (irritability) and E2 (self-destructive or reckless behaviours) symptoms. Unlike the other symptoms included within DSM-5's criterion E that reflect thoughts, feelings, and passive experiences, E1 and E2 represent self-initiating aggressive behaviours. Subsequent to the proposal of the two 6-factor models, researchers proposed a 7-factor Hybrid model which combined the key features of both 6-factor models. The Hybrid model included the division of the hyperarousal factor into anxious and dysphoric arousal, as in the Dysphoric Arousal model from the DSM-IV literature; the separation of the negative affect from the reduced positive affect, as in the Anhedonia model; and the separation of the externalizing behaviours symptoms from the dysphoric arousal factor as in the Externalizing Behaviours model (Armour, 2015; Armour et al., 2015).

Although all three models have to date garnered empirical support, the 7-factor Hybrid model has received support from the US samples of veterans (Armour et al., 2015; Bovin et al., 2015), college students (Armour, Contractor, Shea, Elhai, & Pietrzak, 2016; Armour et al., 2015), an online sample of trauma-exposed adults (Seligowski & Orcutt, 2015), and adult psychiatric outpatients (Zelazny & Simms, 2015). Support for the model has also been found in a nationally representative sample of Australian adults (Carragher et al., 2016), in a sample of Philippine young adult survivors of a typhoon (Mordeno, Carpio, Nalipay, & Saavedra, 2016), and in a sample of Chinese adolescent survivors of an earthquake (Wang et al., 2015). The construct validity of the Hybrid model has also been examined with studies finding differential associations between the factors of the model and a broad range of external constructs (e.g., psychiatric comorbidities, posttraumatic cognitions, anger, functioning, or quality of life; Armour, Contractor et al., 2016; Carragher et al., 2016; Mordeno et al., 2016; Pietrzak et al., 2015; Seligowski & Orcutt, 2015; Wang et al., 2015; Zelazny & Simms, 2015). However, in the nationally representative sample of Australian adults, Carragher et al. (2016) did not find significant differences in model fit between Anhedonia and Hybrid models.

So far, there have only been three studies examining PTSD's latent structure using DSM-5 symptomatology conducted in Europe, and these included samples from Armenia (Demirchyan, Goenjian, & Khachadourian, 2014), Northern Ireland (Armour, Contractor, Palmieri, & Elhai, 2014), and Norway (Hafstad, Dyb, Jensen, Steinberg, & Pynoos, 2014). In this regard, some authors have pointed out the need for future studies that could assess the dimensional structure of PTSD symptoms in other geographic regions (Armour et al., 2015; Armour, Műllerová et al., 2016; Seligowski & Orcutt, 2015).

## Objective

This study aimed to examine the underlying dimensionality of DSM-5 PTSD symptoms in a Spanish sample of traumatized adults. To the best of our knowledge, this is the first study conducted on this topic in this geographic region, and also the first study using the Global Assessment Stress Scale 5, a Spanish self-report measure of posttraumatic symptoms (Crespo, Gómez, & Soberón, 2017). We tested the fit of six PTSD models, including the 4-factor DSM-5 model, a DSM-5 version of the 4-factor Dysphoria model, a DSM-5 version of the 5-factor Dysphoric arousal model, the 6-factor Anhedonia model, the 6-factor Externalizing Behaviours model, and the 7-factor Hybrid model. Based on the existing studies, it was hypothesized that the 7-factor Hybrid model will provide the best fit to the data. Considering that most studies on PTSD's latent structure were conducted with US samples and have assessed the PTSD symptoms with the PCL-5 instrument (Weathers et al., 2013), the current study would extend the existing literature by examining PTSD's dimensional structure in another cultural context and using a different measuring instrument.

## Method

### Participants

Participants (*n=*165) were recruited among individuals seeking treatment from several trauma service providers in the Madrid area of Spain. These included services for battered women, victims of rape or sexual abuse, and the judicial office for assistance to victims in courts and police stations^1^. Inclusion criteria for the study were as follows: (1) exposure to a traumatic event involving death, a life-threatening situation or injury, by directly experiencing the event, witnessing it, or learning that it had occurred to a beloved person; (2) the event must have occurred at least 1 month before the assessment; (3) participants must be aged 18 or older; and (4) they must be fluent in Spanish. Exclusion criteria included (1) current psychosis, (2) cognitive impairment, and (3) substance intoxication at the time of assessment. Professionals of the services selected the service users that fit the criteria between January and May 2015. All the participants provided informed consent and were evaluated by a qualified psychologist in a single session. The study was approved by the Ethics Committee of the Complutense University of Madrid.

### Variables and instruments

PTSD symptoms and trauma history were assessed using the Global Assessment of Posttraumatic Stress Scale 5 (EGEP-5; in Spanish: Evaluación Global de Estrés Postraumático – 5; Crespo et al., 2017). The EGEP-5 was designed as a Spanish self-report measure of posttraumatic stress symptoms as they are outlined in the DSM-5 and to provide both the probable PTSD diagnosis and the symptom severity scores. The EGEP consists of the following three sections:

1. *Events*: This section includes a checklist of 10 traumatic events and an additional open answer trauma question. Individuals are asked to indicate which of these events they have directly experienced, witnessed, or learnt of happening to a close relative or a friend at some point in their lives. Individuals are also asked to choose their most disturbing event and provide its brief description. All the subsequent questions in the EGEP-5 are asked in relation to the most disturbing event. This section also includes 14 items querying the severity and timing of the event, individual's feelings, and the event's implications (e.g., serious injury, death of others, life-threatening potential, and gruesome scenes).
2. *Symptoms*: This section includes the 20 DSM-5 PTSD symptoms (5 for intrusion, 2 for avoidance, 7 for negative alterations in cognitions and mood, and 6 for alterations in arousal and reactivity) and 3 items querying the presence of dissociative symptoms. The participants were asked to indicate whether they have experienced each symptom in the previous month and, if so, the degree of discomfort that it caused them on a 0–4 scale (0=no discomfort; 4=extreme). The two final items are used to rate the duration of the symptoms and their onset.
3. *Functioning*: This section uses seven items (Yes/No) to assess the associated impairment in different areas of life.

Individuals are given a probable diagnosis of the DSM-5 PTSD if they endorse at least one intrusion item, at least one avoidance item, two or more NACM items, two or more AAR items, and two or more impairment items. The PTSD severity is calculated by adding up the scores of the 20 PTSD symptoms. The scores range from 0 to 80, with higher scores indicating more severe symptomatology.

The EGEP-5 demonstrated good internal consistency in the current sample (Cronbach's alpha=0.91). The original EGEP (Crespo & Gómez, 2012), which was based on the DSM-IV symptoms of PTSD, has been found to be highly correlated with external constructs of depression, anxiety, and overall psychopathology that are often comorbid with PTSD. Moreover, the diagnostic performance analyses, taking the Composite International Diagnostic Interview—CIDI (World Health Organization [WHO], 1990) as the “gold standard,” yielded the following indices: sensitivity of 91%, specificity of 75%, positive predictive value of 89%, negative predictive value of 78%, accuracy of 86.1%, Youden index of 0.66, and the Kappa index of 0.67 (*p<*0.001).

### Data analysis

The analyses focused on the 20 EGEP-5 items that assess the severity of the DSM-5 PTSD symptoms. Missing data were estimated in Mplus 6.12 (Muthén & Muthén, 2010, 1998–2011) using the robust maximum likelihood estimation. Mplus was also used to conduct the confirmatory factor analyses (CFAs) in order to determine and compare the fit of the six PTSD models to the current data. The MLR estimation method was applied to correct for non-normality. In all of the measurement models estimated, error covariances and factor variances were fixed to 0 and 1, respectively, to scale the factors within the specified models. Goodness-of-fit indices were obtained for each of the specified models and included the comparative fit index (CFI), the Tucker–Lewis Index (TLI), the root mean square error of approximation (RMSEA), and the standardized root mean square residual (SRMR). According to Hu and Bentler (1998), excellent fit is achieved with CFI/TLI above 0.95 and RMSEA/SRMR below 0.60, and adequate fit is achieved with CFI/TLI above 0.90 and RMSEA/SRMR below 0.80. To compare nested models, we used the chi-square difference tests with a correction factor (Muthèn & Muthèn, 2010). A significant (*p<*0.05) chi-square indicates that the model with the lower chi-square value provides a better fit, whereas a non-significant (*p*≥0.05) chi-square suggests that the models do not differ significantly in their fit (Armour, Mu˝llerová et al., 2016). To compare the non-nested models, we calculated the Bayesian information criterion (BIC) and the Akaike information criterion (AIC). A BIC difference of 6–10 indicates strong support and a difference greater than 10 indicates very strong support for the model with the lower BIC value (Raftery, 1995). According to Akaike (1987), the model with a lower AIC value is preferred. Standardized factor loadings of competing models and factor correlations of the best-fitting model were also examined.

## Results

### Sample characteristics

The mean age of participants was 38.21 (SD=11.71) years, ranging from 18 to 79, and 78.8% were female.

Participants experienced, on average, 6.08 (SD=3.82) traumatic events at some point in their lives. This included events that were directly experienced (*M=*3.3; SD=1.53), witnessed (*M=*1.01; SD=1.83), and heard of as happening to a close person (*M=*1.77; SD=2.11). A wide range of traumatic events was reported, with the most frequently nominated worst traumatic events being physical violence, harassment, accidents, and rape or sexual abuse (see Table 1). Most of the participants (89.8%) described their worst event as severe or extreme. A total of 41.2% of participants reported that the event first occurred 3 months before the assessment, and 51.5% reported repeated exposure to the event. For 87.3% of victims, the event involved gruesome scenes, for 72.7% it involved threats to physical integrity, and for 59.4% it included life-threatening situations.

**Table 1:** Descriptive statistics of traumatic events and PTSD symptoms according to EGEP-5 scores

	*n=*165
Type of trauma *n* (%)	
Natural disasters	2 (1.2)
Accidents	30 (18.1)
Combat or war	1 (0.6)
Rape or sexual abuse	16 (9.6)
Harassment	35 (21.1)
Physical violence	61 (36.7)
Terrorism or torture	11 (6.6)
Death of a beloved person	9 (5.4)
Other	1 (0.6)
EGEP-5 PTSD symptoms severity *M* (SD)	
Total score (0–80)	37.21 (17.9)
Intrusion (0–20)	10.30 (5.6)
Avoidance (0–8)	4.09 (2.7)
Negative alterations in cognitions and mood (0–28)	12.26 (6.8)
Alterations in arousal and reactivity (0–24)	10.41 (5.7)
Proportion of participants meeting the DSM-5 PTSD criteria *n* (%)	
PTSD	
B – Intrusion	111 (67.3)
C – Avoidance	158 (95.8)
D – Negative alterations in cognitions and mood	142 (86.1)
E – Alterations in arousal and reactivity	140 (84.8)
F – Duration	148 (89.7)
G – Functional impairment	157 (95.2)
	145 (87.9)

Descriptive statistics of the EGEP-5 PTSD symptoms are presented in Table 1. Based on the DSM-5, a total of 111 (67.3%) participants met the criteria for a probable PTSD diagnosis, with criterion B (intrusive symptoms) being the most frequently endorsed symptom in this sample (95.8%). In addition, 86.1% of respondents met criterion C (avoidance symptoms), 84.8% met criterion D (NACM symptoms), and 89.7% met criterion E (AAR symptoms). Only four respondents (2.4%) did not meet the criteria for positively endorsing any of the DSM-5 PTSD symptom clusters. Finally, 95.2% of respondents met the duration criterion (F) and 87.9% reported functional impairment (criterion G). Regarding the PTSD symptom severity (see Table 1), the mean EGEP-5 total score and the mean subscale scores were all indicative of mild PTSD severity.

### Dimensional structure of PTSD

Table 2 reports the Goodness-of-fit indices for the six competing PTSD models. According to Hu and Bentler's (1998) criteria (adequate fit is achieved with CFI/TLI above 0.90 and RMSEA/SRMR below 0.80), all models provided adequate fit, with some approaching excellent fit (CFI/TLI above 0.95 and RMSEA/SRMR below 0.60).

**Table 2:** Confirmatory factor analyses fit indices for the six PTSD models

Model	χ^2^	df	CFI	TLI	RMSEA (90% CI)	SRMR	BIC	AIC
DSM-5	251.33	164	0.92	0.91	0.057 (0.042–0.070)	0.060	10767.47	10562.48
Dysphoria	243.94	164	0.93	0.92	0.054 (0.039–068)	0.061	10758.37	10553.38
Dysphoric arousal	229.90	160	0.94	0.93	0.051 (0.036–0.066)	0.058	10762.35	10544.94
Externalizing behaviours	219.50	155	0.94	0.93	0.050 (0.034–0.065)	0.054	10776.53	10543.59
Anhedonia	214.89	155	0.95	0.94	0.048 (0.031–0.063)	0.055	10770.15	10537.20
Hybrid	203.62	149	0.95	0.94	0.047 (0.029–0.063)	0.052	10788.40	10536.82

CFI, comparative fit index; TLI, Tucker–Lewis index; RMSEA, root mean square error of approximation; SRMR, standardized root mean square residual; BIC, Bayesian information criterion; AIC, Akaike information criterion.

The comparisons of the nested models showed that the 6-factor Externalizing Behaviours model provided a significantly better fit than the 4-factor DSM-5 model (Δχ^2^(9)=30.21, *p<*0.001) and the 4-factor Dysphoria model (Δχ^2^(9)=24.08, *p=*0.004). No significant differences in model fit were found between the 6-factor Externalizing Behaviours model and the 5-factor Dysphoric Arousal model (Δχ^2^(5)=10.50, *p=*0.062). The 6-factor Anhedonia model provided a significantly better fit than the 4-factor DSM-5 model (Δχ^2^(9)=32.70, *p<*0.001), the 4-factor Dysphoria model (Δχ^2^(9)=27.22, *p=*0.001), and the 5-factor Dysphoric Arousal model (Δχ^2^(5)=14.02, *p=*0.015). When comparing the non-nested models, it was found that the 4-factor Dysphoria model provided a better fit than the 4-factor DSM-5 model, as evidenced by a 9.1-point BIC difference and a lower AIC value, and the 6-factor Anhedonia model provided a better fit than the 6-factor Externalizing Behaviours model, as evidenced by a 6.38-point BIC difference and a lower AIC value. The 7-factor Hybrid model provided a significantly better fit than the 4-factor DSM-5 model (Δχ^2^(15)=45.90, *p<*0.001), the 4-factor Dysphoria model (Δχ^2^(15)=39.00, *p=*0.001), the 5-factor Dysphoric Arousal model (Δχ^2^(11)=25.64, *p=*0.007), and the 6-factor Externalizing Behaviours model (Δχ^2^(6)=14.97, *p=*0.020). However, no significant differences in model fit were found between the 7-factor Hybrid model and the 6-factor Anhedonia model (Δχ^2^(6)=11.27, *p=*0.080).

Standardized factor loadings for the six competing models are presented in Table 3. The majority of these factor loadings were moderate to high, ranging from 0.57 to 0.81, and were highly similar across models. Nevertheless, the factor loadings for the trauma-related amnesia, blame of self or others, and reckless behaviour symptoms were consistently lower across all models.

**Table 3:** Standardized factor loading for the six competing models

DSM-5 symptom	DSM-5	DSM-5 Dysphoria	DSM-5 Dysphoric arousal	Externalizing behaviours	Anhedonia	Hybrid
1. Intrusive thoughts	0.80 (I)	0.80 (I)	0.80 (I)	0.80 (I)	0.80 (I)	0.80 (I)
2. Nightmares	0.62 (I)	0.62 (I)	0.62 (I)	0.62 (I)	0.62 (I)	0.62 (I)
3. Flashbacks	0.72 (I)	0.71 (I)	0.72 (I)	0.72 (I)	0.72 (I)	0.72 (I)
4. Emotional cue reactivity	0.81 (I)	0.81 (I)	0.81 (I)	0.81 (I)	0.81 (I)	0.81 (I)
5. Physiological cue reactivity	0.81 (I)	0.81 (I)	0.81 (I)	0.81 (I)	0.81 (I)	0.81 (I)
6. Avoidance of thoughts	0.77 (A)	0.78 (A)	0.77 (A)	0.77 (A)	0.79 (A)	0.79 (A)
7. Avoidance of reminders	0.76 (A)	0.75 (A)	0.76 (A)	0.76 (A)	0.74 (A)	0.74 (A)
8. Trauma-related amnesia	0.34 (NACM)	0.32 (D)	0.34 (NACM)	0.35 (NACM)	0.33 (NACM)	0.35 (NA)
9. Negative beliefs	0.59 (NACM)	0.57 (D)	0.57 (NACM)	0.58 (NACM)	0.61 (NACM)	0.60 (NA)
10. Blame of self or others	0.36 (NACM)	0.35 (D)	0.36 (NACM)	0.37 (NACM)	0.37 (NACM)	0.38 (NA)
11. Negative trauma-related emotions	0.69 (NACM)	0.68 (D)	0.68 (NACM)	0.68 (NACM)	0.72 (NACM)	0.70 (NA)
12. Loss of interest	0.67 (NACM)	0.69 (D)	0.68 (NACM)	0.67 (NACM)	0.72 (An)	0.72 (An)
13. Detachment	0.64 (NACM)	0.63 (D)	0.64 (NACM)	0.65 (NACM)	0.66 (An)	0.66 (An)
14. Restricted affect	0.71 (NACM)	0.70 (D)	0.71 (NACM)	0.71 (NACM)	0.75 (An)	0.75 (An)
15. Irritability	0.64 (AAR)	0.67 (D)	0.68 (DA)	0.73 (EB)	0.67 (DA)	0.72 (EB)
16. Self-destructive/reckless behaviour	0.31 (AAR)	0.25 (AAR)	0.33 (DA)	0.37 (EB)	0.32 (DA)	0.38 (EB)
17. Hypervigilance	0.67 (AAR)	0.77 (AAR)	0.78 (AA)	0.78 (AA)	0.78 (AA)	0.79 (AA)
18. Exaggerated startle response	0.64 (AAR)	0.75 (AAR)	0.76 (AA)	0.76 (AA)	0.76 (AA)	0.76 (AA)
19. Difficulty concentrating	0.63 (AAR)	0.65 (D)	0.66 (DA)	0.65 (DA)	0.67 (DA)	0.65 (DA)
20. Sleep disturbance	0.68 (AAR)	0.66 (D)	0.68 (DA)	0.70 (DA)	0.68 (DA)	0.70 (DA)

I, intrusion; A, avoidance; NACM, negative alterations in cognition and mood; AAR, alterations in arousal and reactivity; D, dysphoria; DA, dysphoric arousal; AA, anxious arousal; EB, externalizing behaviours; An, anhedonia; NA, negative affect.

Table 4 presents the factor intercorrelations for the 6-factor Anhedonia model and the 7-factor Hybrid model. All factors were moderately to highly correlated, with the correlations ranging from 0.65 to 0.93 for the Anhedonia model and 0.55 to 0.90 for the Hybrid model.

**Table 4:** Correlations between anhedonia model factors and hybrid model factors (*n=*165)

	I	A	NA	An	EB	AA	DA
I		0.80	0.69	0.59	—	0.71	0.73
A			0.67	0.71	—	0.70	0.65
NA				0.86	—	0.86	0.91
An					—	0.66	0.93
AA					—		0.74
DA					—		
I		0.80	0.69	0.59	0.56	0.71	0.81
A			0.67	0.71	0.55	0.70	0.68
NA				0.86	0.93	0.87	0.86
An					0.90	0.66	0.89
EB						0.64	0.90
AA							0.77
DA							

I, Intrusion; A, Avoidance; NA, Negative affect; An, anhedonia; EB, externalizing behaviours; AA, anxious arousal; DA, dysphoric arousal.

## Discussion

This study was the first to examine the underlying dimensionality of DSM-5 PTSD in a Spanish clinical sample exposed to a range of traumatic events. Six different competing DSM-5 PTSD models were examined. Based on the Goodness-of-fit indices, all models provided good fit to the data, with the 7-factor Hybrid model providing the best fit to the data based on a slightly lower TLI and RMSEA compared with the next best-fitting model, the Anhedonia model. However, the statistical comparisons of nested models revealed that the 7-factor Hybrid model did not fit significantly better than the 6-factor Anhedonia model. This result diverges from most of the extant literature assessing the fit of the Hybrid model (Armour, Contractor et al., 2016; Armour et al., 2015; Bovin et al., 2015; Mordeno et al., 2016; Seligowski & Orcutt, 2015; Zelazny & Simms, 2015), but it is consistent with Carragher et al.'s (2016) and Wang et al.'s (2015) studies, which also reported similar values of fit indices across the models and found no significant differences in model comparisons between the Anhedonia and Hybrid models. As Carragher et al. (2016) suggested, this divergence with previous studies may be related to sample characteristics. However, we used a clinical sample, whereas Carragher et al. (2016) used a national sample. Nevertheless, even though their sample consisted of mainly women, their proportion was not so large (56% vs. 78.8%). In addition, the type of trauma experience could be another potential explanation for the disparity. In most of the previous studies where the Hybrid model emerged as the best-fitting model, the most endorsed traumatic events were a family member's or close friend's death (Armour, Contractor et al., 2016; Armour et al., 2015) and natural disasters (Mordeno et al., 2016; Wang et al., 2015), whereas in our study the most frequently reported traumatic event was physical violence. Unfortunately, Carragher et al. (2016) did not report information on the type of trauma experienced.

The finding that the comparison of the two 6-factor models (Anhedonia and Externalizing behaviours) indicated a better fit for the Anhedonia model, together with the fact that the main difference between the Hybrid model and the Anhedonia model is the separation of the externalizing behaviours symptom cluster in the former, suggests that in the current sample the separation of the externalizing behaviours symptoms into their own symptom cluster cannot be justified. One potential explanation could be the greater proportion of females in our sample (78.8%), which could suggest the moderating effects of gender on model fit. In a recent review, Armour, Mu˝llerová et al. (2016) discussed gender as a potential moderator of model fit in the DSM-IV studies of PTSD's latent structure, although they noted that the findings so far have been equivocal. With regard to the DSM-5 models, Tsai et al. (2014) reported that the 6-factor Externalizing Behaviours model provided a better fit than the 5-factor Dysphoric Arousal model in their sample of female veterans; however, the fit indices for the Externalizing Behaviours model were much lower in the female subsample than in the total sample, and they were almost identical in the Externalizing Behaviours and the Dysphoric Arousal models in the female sample. Therefore, it is possible that the externalizing behaviours symptoms (i.e., self-destructive/reckless behaviour and irritability) do not play a major role in the PTSD diagnoses in females. In fact, Carmassi et al. (2013) found significantly higher rates of externalizing behaviours symptoms in males (63.6%) than in females (20.5%). Moreover, they reported that these symptoms were crucial in 31.17% of the PTSD diagnoses in males but only in 3.94% of the diagnoses in females.

In summary, the 6-factor Anhedonia model and the 7-factor Hybrid model provided a superior fit to the data compared with the 4-factor and 5-factor models— a finding that is reported consistently across studies (Armour, Contractor et al., 2016; Armour et al., 2015; Bovin et al., 2015; Carragher et al., 2016; Mordeno et al., 2016; Seligowski & Orcutt, 2015; Wang et al., 2015; Zelazny & Simms, 2015). However, the findings have not supported the suggestion of a separate externalizing behaviour factor referring to self-initiating aggressive behaviours that may reflect deficits in emotion regulation and impulse control (Tsai et al., 2014). On the other hand, since the models that included the differentiation between the anxious arousal and dysphoric arousal symptoms fit the data significantly better than the models in which these symptoms were grouped together, the findings yield further evidence in support of the dysphoric and anxious arousal factors as separate constructs within PTSD (Elhai et al., 2011). Moreover, the finding that the models that differentiated between the symptoms of negative affect and anhedonia fit better than the models that grouped these symptoms supports their uniqueness as proposed by Liu et al. (2014).

The results contradict our main hypothesis about the superiority of the Hybrid model over all alternative models. In this regard, in addition to the aforementioned considerations, the fact that some researchers suggest a possible effect of population type, trauma type, and PTSD measures on the model fit must be highlighted (Elhai & Palmieri, 2011). Furthermore, the high rate of probable PTSD diagnosis in our sample (67.3%) when compared to the rates in previous studies that assessed this model (ranging from 4.02 to 45.9%; Armour, Contractor et al., 2016; Armour et al., 2015; Carragher et al., 2016; Mordeno et al., 2016; Seligowski & Orcutt, 2015; Wang et al., 2015; Zelazny & Simms, 2015) might have contributed, to some extent, to the contradiction of our hypothesis. In fact, Biehn, Elhai, Fine, Seligman, and Richardson (2012) examined differences in PTSD's dimensional structure between Canadian veterans with and without a PTSD diagnosis and found that the models demonstrated a better fit in the sample who did not have a PTSD diagnosis. Thus, the authors pointed out the importance of assessing PTSD models fit among clinical samples to ensure an accurate representation of the disorder.

In line with the majority of previous studies (Armour, Contractor et al., 2016; Armour et al., 2015; Hafstad et al., 2014; King et al., 1998; Liu et al., 2014; Miller et al., 2013; Seligowski & Orcutt, 2015; Simms et al., 2002; Wang et al., 2015), the smallest factor loading across all models was for the trauma-related amnesia item, possibly suggesting that it is not a good indicator of posttraumatic psychopathology. Similarly, the low factor loadings of the new DSM-5 reckless behaviour symptom across all the models are congruent with the results of some previous DSM-5 studies (Hafstad et al., 2014; Liu et al., 2014; Miller et al., 2013). According to Hafstad et al. (2014), this symptom could be more relevant to certain subgroups of trauma survivors, such as victims of certain types of traumatic events (e.g., combat veterans). The endorsement of this symptom could also be influenced by the type of PTSD instrument used, the cultural influences (i.e., the social tolerance for reckless behaviour), and the associated social desirability. Finally, in line with the results reported by Wang et al. (2015), the factor loadings of the item related to blame of self or others were also considerably low. Future research may wish to determine the role of the externalizing behaviours symptoms in PTSD, with particular attention to the potential moderating effects of gender and other sample characteristics.

Moreover, as Armour, Mu˝llerová et al. (2016) pointed out, CFA findings do not inform about the number of symptoms from each cluster that would be needed for a PTSD diagnosis. Further research is therefore needed to examine whether positive endorsements of symptoms from all six (in case of the 6-factor models) or seven (in case of the Hybrid model) symptom clusters would be needed for a diagnosis of PTSD, since several studies have shown that changes in diagnostic criteria and dimensional structure have implications to PTSD prevalence rates estimations (e.g., Hansen, Hyland, Armour, Shevlin, & Elklit, 2015; Schaal, Koebach, Hinkel, & Elbert, 2015).

The current findings must be interpreted with some caution in view of certain study limitations. Firstly, as Armour, Műllerová et al. (2016) noted, the small number of indicators across factors (mainly in the Hybrid model) could have skewed the CFAs results, and this could especially be problematic considering our small sample size. Of note, the DSM-5 model comprised symptom factors consisting of only two items. Secondly, PTSD symptoms were assessed with a single self-report measure. Different measures of PTSD symptoms, including a clinical interview, may offer different findings in model fit (Elhai & Palmieri, 2011) and increase the validity of PTSD's dimensional structure conclusions. Thirdly, the generalizability of the results is limited as most participants were females. Further studies using clinical samples where both genders and different traumatic events are represented equally are needed. In addition, this would allow for accurate conclusions regarding gender differences in PTSD models fit and symptom expression to be drawn. Finally, the current study did not examine the construct validity of the best-fitting models through the analysis of associations between symptom clusters and external variables of comorbid symptoms (e.g., anxiety) or key outcomes (e.g., quality of life).

Despite these limitations, this was the first study to be conducted with a Spanish clinical sample exposed to a range of traumatic events, and it provides further support for the 6- and 7-factor models of the DSM-5 PTSD. In line with some previous studies, our findings raise important issues about the externalizing behaviour symptoms, which should be addressed in future studies.

## Footnotes

1 Sample was recruited through the following services: Beccaria Association for Assistance to Victims, Federation of Progressive Women, Centre for the Assistance to Victims of Sexual Aggressions (CAVAS), Services for Assistance to Victims of the Spanish Justice Ministry, Service for Assistance to Terrorism Victims of the National Audience, and Services for Equality of the Madrid City Hall.
